# Supercharged eGFP-TRAIL Decorated NETs to Ensnare and Kill Disseminated Tumor Cells

**DOI:** 10.1007/s12195-020-00639-8

**Published:** 2020-08-06

**Authors:** Thong M. Cao, Michael R. King

**Affiliations:** Department of Biomedical Engineering, Vanderbilt University, Nashville, TN 37235 USA

**Keywords:** NETosis, Neutrophil, Metastasis, TRAIL, Apoptosis, Protein engineering

## Abstract

**Background:**

NETosis is an innate immune response elicited by activated neutrophils to fight microbial infections. Activated neutrophils release DNA fibers decorated with anti-microbial proteins called neutrophil extracellular traps (NETs) into the extracellular space to trap and kill surrounding microbes.

**Methods:**

Here, we show that tumor-derived IL-8 released by cancer cells also activates the release of NETs. Until now, there have been no existing technologies that leverage NETs as an anti-tumor drug delivery vehicle. In this study, we demonstrate the re-engineering of neutrophils to express an apoptosis-inducing chimeric protein, supercharged eGFP-TRAIL, on NETs that can ensnare and kill tumor cells while retaining their anti-microbial capabilities.

**Results:**

We observed significant TRAIL-induced apoptosis in tumor cells captured by TRAIL-decorated NETs.

**Conclusions:**

This work demonstrates NETs as a promising technology to deliver protein in response to local cytokine signals.

## Introduction

Neutrophils play a significant role in all stages of tumorigenesis, from the initial genotoxic insult, to metastasis to distant organs. Chronic inflammation drives neutrophils to release mutagenetic agents, including reactive oxygen species (ROS) and hypochlorous acid (HOCl) that induce DNA damage and mutagenicity on surrounding cells.^2^,^7^,^9^ Neutrophils comprise a significant percentage of white blood cells that infiltrate the tumor microenvironment.^8^ In that setting, infiltrating neutrophils continue to promote tumor development by secreting pro-inflammatory and pro-angiogenesis chemokines and cytokines such as matrix metallopeptidase 9 (MMP9) and interleukin 6 (IL-6).^1^,^15^,^19^ Tumor metastasis* via* hematogenous dissemination involves circulating tumor cells (CTCs) shedding from the primary tumor site and reaching distant organs through the circulatory system. In a recent study, neutrophils have been found to support CTC survival during hematogenous dissemination.^18^ Furthermore, neutrophils have been identified as the main driver in establishing the pre-metastatic microenvironment in several mouse breast cancer models.^20^

NETosis is a unique form of innate immune response elicited primarily by neutrophils to combat microbial infections.^3^ In the presence of antigens, neutrophils undergo NETosis by following a program of cell death and releasing condensed DNA fibers decorated with cationic antimicrobial proteins, collectively called NETs, into the extracellular space.^16^ Tumor cells release tumor-derived interleukin 8 (IL-8), a potent neutrophil chemoattractant, to increase tumor growth and metastatic potential by: (i) promoting tumor neovascularization, and (ii) inducing infiltrating neutrophils to release pro-metastatic enzymes.^4^

## Results

**Figure 1:**
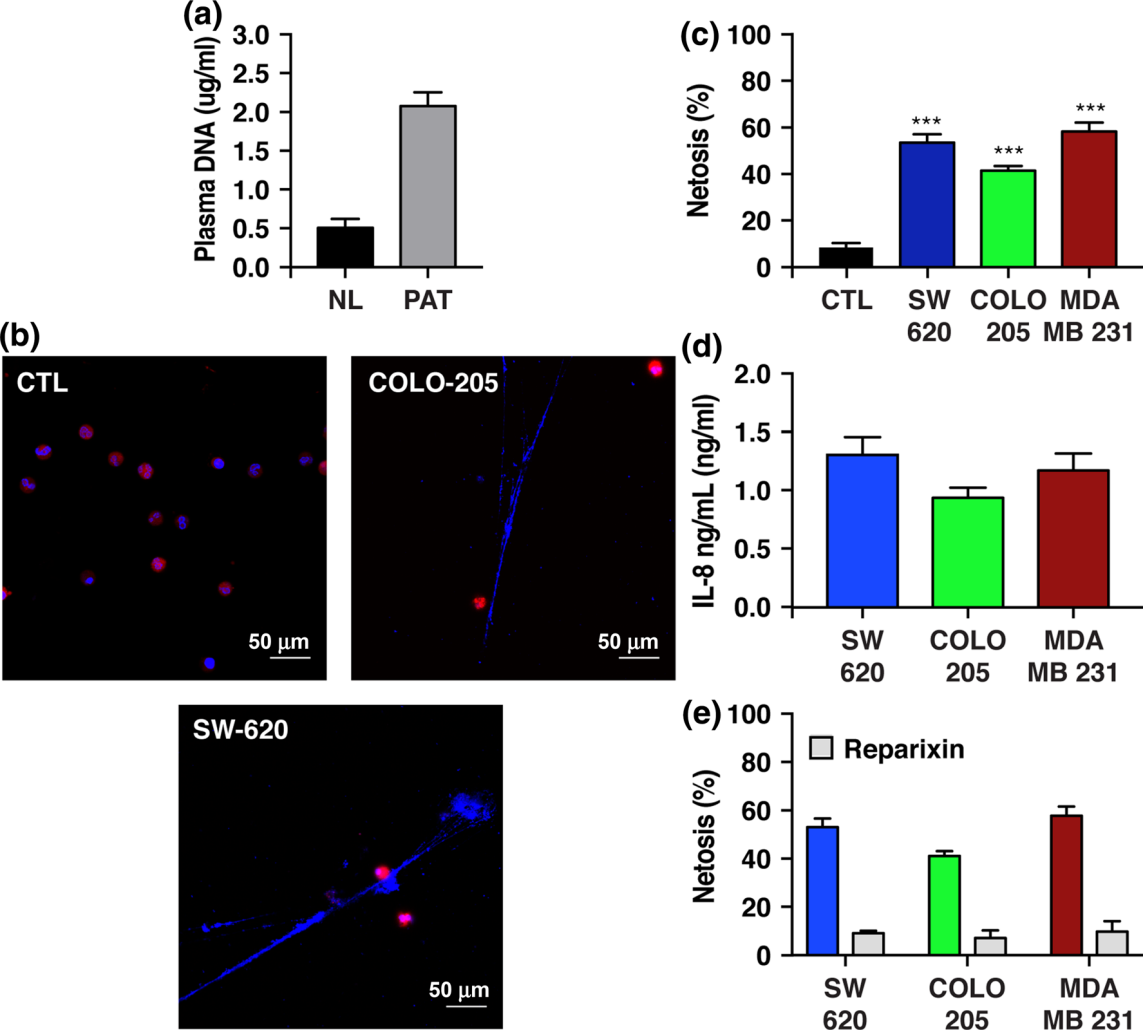
Neutrophils undergo spontaneous NETosis in the presence of tumor cells. (a) cfDNA plasma DNA level of normal donors (ND) and cancer patients (PAT). (b) Confocal images of neutrophils that underwent NETosis in conditioned media. (CTL)—control group, COLO-205 and SW-620 are conditioned media collected after 48 h from these indicated cell lines. (c) NETosis level of neutrophils after culturing in conditioned media for 24 h. (d, e) Extracellular tumor-derived IL-8 levels in conditioned media. Reparixin concentration was 20 μg/mL.

Initially, NETosis was found to specifically occur in the presence of bacterial antigens, but here we show that tumor-derived IL-8 released by tumor cells also elicits NETosis in neutrophils (Figs. 1b and 1c). Blocking the tumor-derived IL-8 pathway with a small molecule, reparixin, returned NETosis back to basal levels (Fig. 1e). Moreover, we quantified cell-free serum DNA levels in cancer patients and found a significant increase in serum DNA level compared to the healthy cohort, suggesting that NETs may also have an elevated presence in the circulatory system in cancer patients (Fig. 1a). These results support previous findings that malignant and non-malignant neutrophils in tumor-bearing mice have increased sensitivity toward undergoing NETosis.^5^ Given data shown here of the ubiquitous presence of neutrophils in all stages of tumor development, we proposed a unique anti-tumor drug delivery system by exploiting the ability of neutrophils to spontaneously undergo NETosis in the presence of cancer cells.

**Figure 2:**
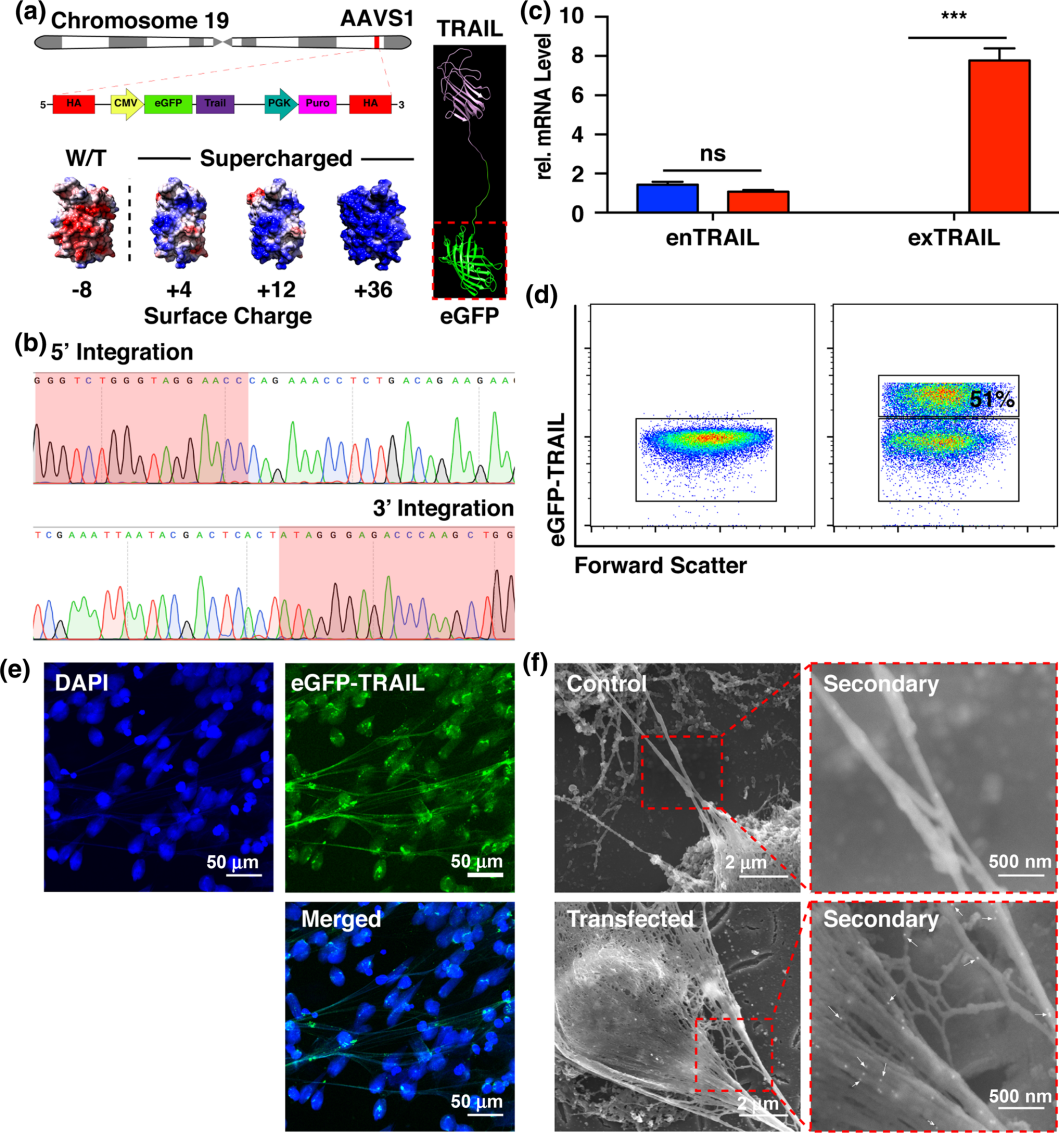
Engineered neutrophils express supercharged eGFP-TRAIL on NETs during NETosis. (a) Schematic of the insertion site, AAVS1, on chromosome 19. Cartoon representation of eGFP-TRAIL chimeric protein. Surface charge of eGFP ranging from − 4 to + 36 (Red—negative charge, Blue—positive charge. (b) DNA sequencing result of genomic DNA isolated from cells positive for eGFP-TRAIL. (c) relative mRNA level of endogenous (enTRAIL) and exogenous (exTRAIL) TRAIL levels. (d) Flow cytometry of cells expressing eGFP-TRAIL 24 h after nucleofection. (e) Confocal images of NETs decorated with eGFP-TRAIL (Blue—DAPI stain, Green—eGFP-TRAIL). (f) Immuno-gold SEM images of neutrophils expressing eGFP-decorated NETs (White arrows—eGFP-TRAIL).

**Figure 3:**
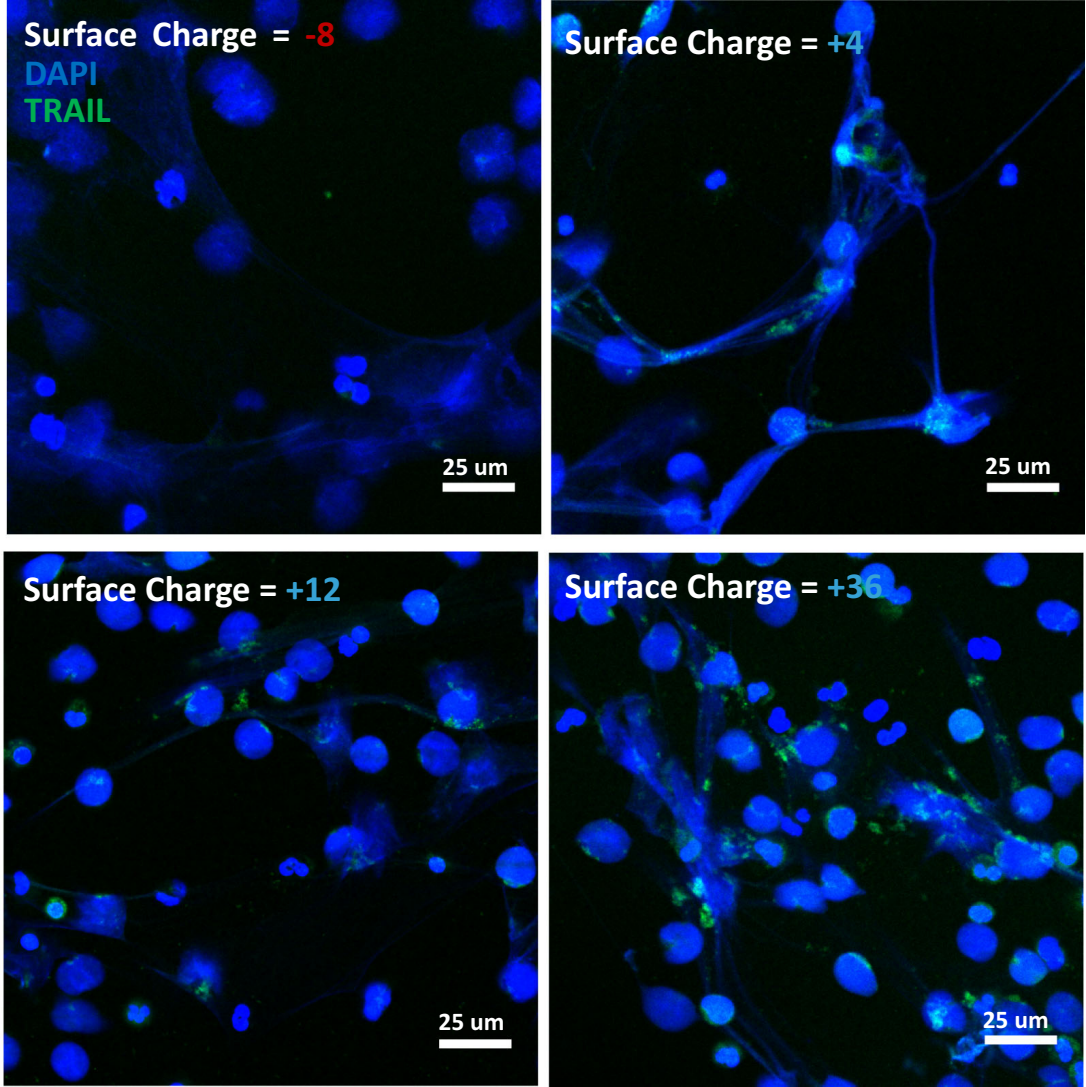
The NET-TRAIL complex is mediated by eGFP surface charge. Micrographs have been pseudocolored and overlaid to visually display the degree of overlap between DNA (in blue) and eGFP-TRAIL (in green) for four different net electrostatic charges of the eGFP protein. Note the lack of eGFP-TRAIL colocalized signal in the (− 8) negatively charged case.

Although NETs have been shown to ensnare cancer cells, they lack the ability to kill cancer cells. More insidiously, trapped tumor cells show increased metastatic potential.^6^ In the current study, we proposed to re-engineer human neutrophils to express NETs decorated with an apoptosis-inducing peptide, TRAIL, that could selectively destroy cancer cells during NETosis. TRAIL is a small cytokine expressed by most cell types that selectively induces apoptosis in tumor cells overexpressing death receptors while sparing healthy cells.^11^,^14^ In this model, we successfully knocked in our gene of interest (GOI), expressing the chimeric protein eGFP-TRAIL, into the safe harbor site AAVS1 on chromosome 19 of the proto-neutrophilic cell line PLB-985 using the CRISPR/Cas system (Figs. 2a and 2b). PLB-985 is a leukemic cell line that can be induced into a neutrophil-like state capable of undergoing NETosis.^12^,^17^ Aside from serving as a fluorescent marker, eGFP serves a more important function by selectively allowing the chimeric protein to electrostatically bind to the DNA fibers of NETs during NETosis (Fig. 2a). By modifying the surface charge of eGFP to become increasingly more positive, we were able to increase its avidity to the negatively-charged DNA fibers in a charge-dependent manner (Fig. 3). The transfected cells stably expressed eGFP-TRAIL-decorated NETs during NETosis (Figs. 2e–2f). mRNA data showed successful expression of the transgene. Immunostaining scanning electron microscopy with TRAIL antibody-conjugated gold nanoparticles of NET DNA fibers revealed that eGFP-TRAIL protein molecules are decorated along the DNA fibers (Fig. 2f).

**Figure 4:**
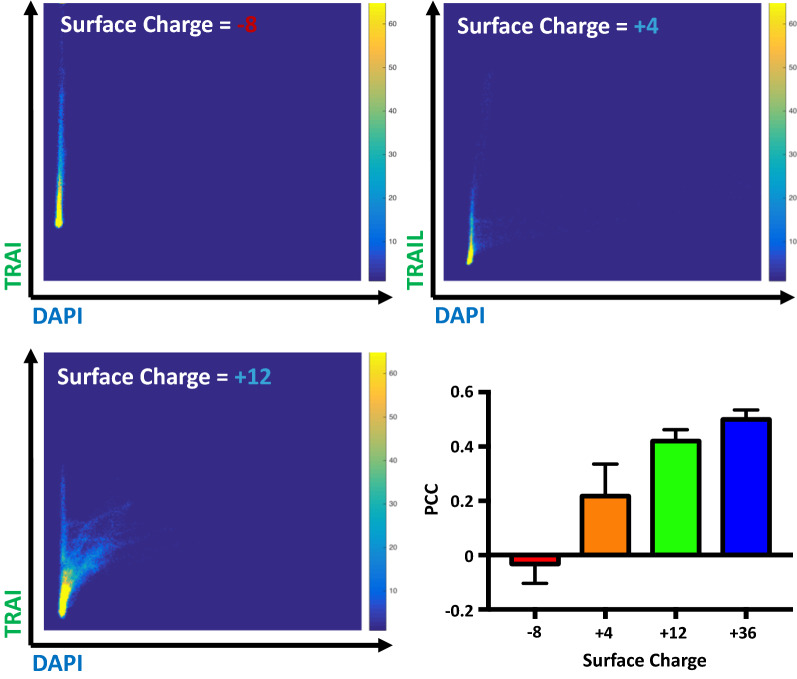
The degree of TRAIL/DAPI(DNA) colocalization is a function of the net surface charge of the eGFP molecule. A Pearson correlation coefficient (PCC) analysis was performed on the fluorescence images, shown in representative cases in Fig. 3, and plotted as a function of the eGFP surface charge.

We examined how the degree of DNA:eGFP-TRAIL association depends on the electrostatic surface charge of the supercharged eGFP molecule. It was expected that the net negative charge of the wildtype eGFP protein would need to be manipulated to become positive to enable association with DNA fibers following exteriorization by the NETosis process. As anticipated, the (− 8) negatively charged eGFP showed no visible colocalization with DNA nets, however the + 4 through + 36 eGFP proteins showed visible colocalization with NETs as evidenced by representative fluorescence micrographs (Fig. 3). When an analysis was performed to yield the scalar Pearson Correlation Coefficient (PCC), we observed PCC to be a monotonically increasing function of increased positive charge of the eGFP molecule, indicative of an increasing degree of pixel correlation (Fig. 4). While the most supercharged version of the eGFP protein (+ 36) would seem to be the optimal formulation for NETosis delivery based on this PCC analysis, it exhibited significant precipitation and aggregation and was not tested further in cellular activity assays.

**Figure 5:**
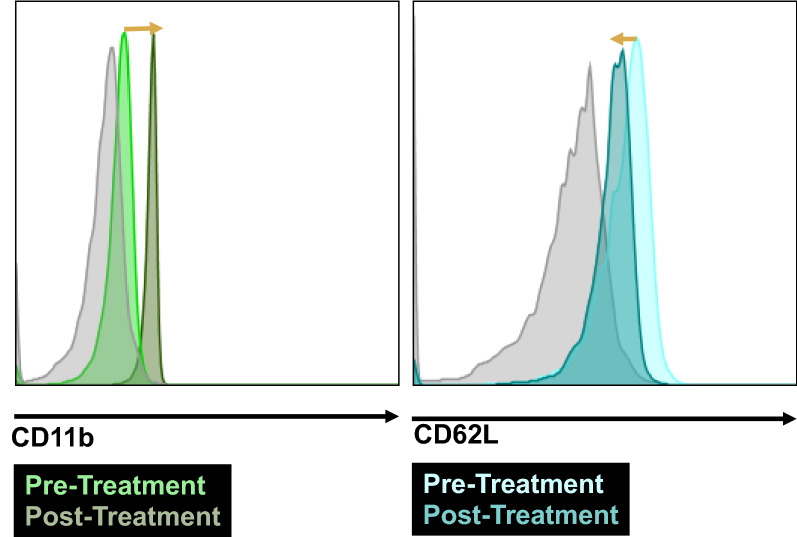
Transfected PLB-985 cells retain neutrophilic traits after differentiation. Flow cytometric analysis shows that fMLP stimulation of the PLP-985 model neutrophils exhibit the correct molecular traits of early neutrophil activation, including an upregulation of active-conformation CD11b (left graph) and a downregulation of surface CD62L (right graph). Gray curve is isotype control.

**Figure 6:**
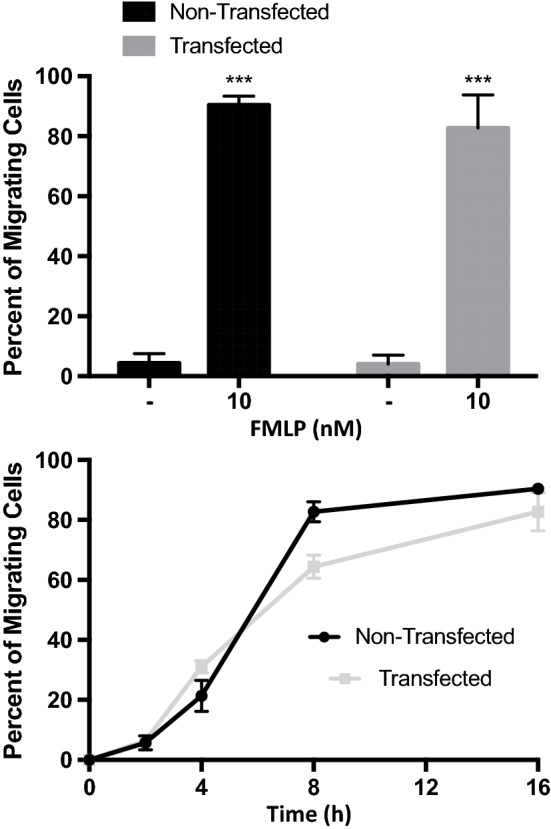
Neutrophil-like characteristics are retained after fMLP treatment. eGFP-TRAIL transfected cells showed a similar percentage of migrating cells in the presence of 10 nM of fMLP chemoattractant in a Boyden chamber assay.

Prior to testing the cytotoxic activity of transfected PLB-985 cells, we performed tests to verify that they retain certain neutrophil-like phenotypes. Two of the earliest, molecular indicators of neutrophil activation* via* the peptide fMLP or inflammatory cytokines are (i) regulation of Mac-1 (CD11b) into an active conformation, and (ii) enzymatic cleavage of L-selectin (CD62L) from the cell surface.^10^,^13^ Both of these responses occur before shape change and other motility-associated functions, and can be easily assayed* via* flow cytometry. Figure 5 shows a rightward shift in staining of active CD11b marker using the conformation-specific monoclonal antibody CBRM1/5, as well as a clear leftward shift in surface L-selectin expression following activation* via* 10 nM of fMLP. As a later readout of activated neutrophil function, Fig. 6 shows a highly significant fraction of migrating PLB-985 cells following activation* via* 10 nM of fMLP, and the percentage of cells migrating through a transwell migration assay was similar between the two groups of cells with and without eGFP-TRAIL transfection. From these data we may conclude that these normal neutrophilic functions of acute inflammation may proceed unimpeded while carrying the NETosis-based delivery vehicle.

**Figure 7:**
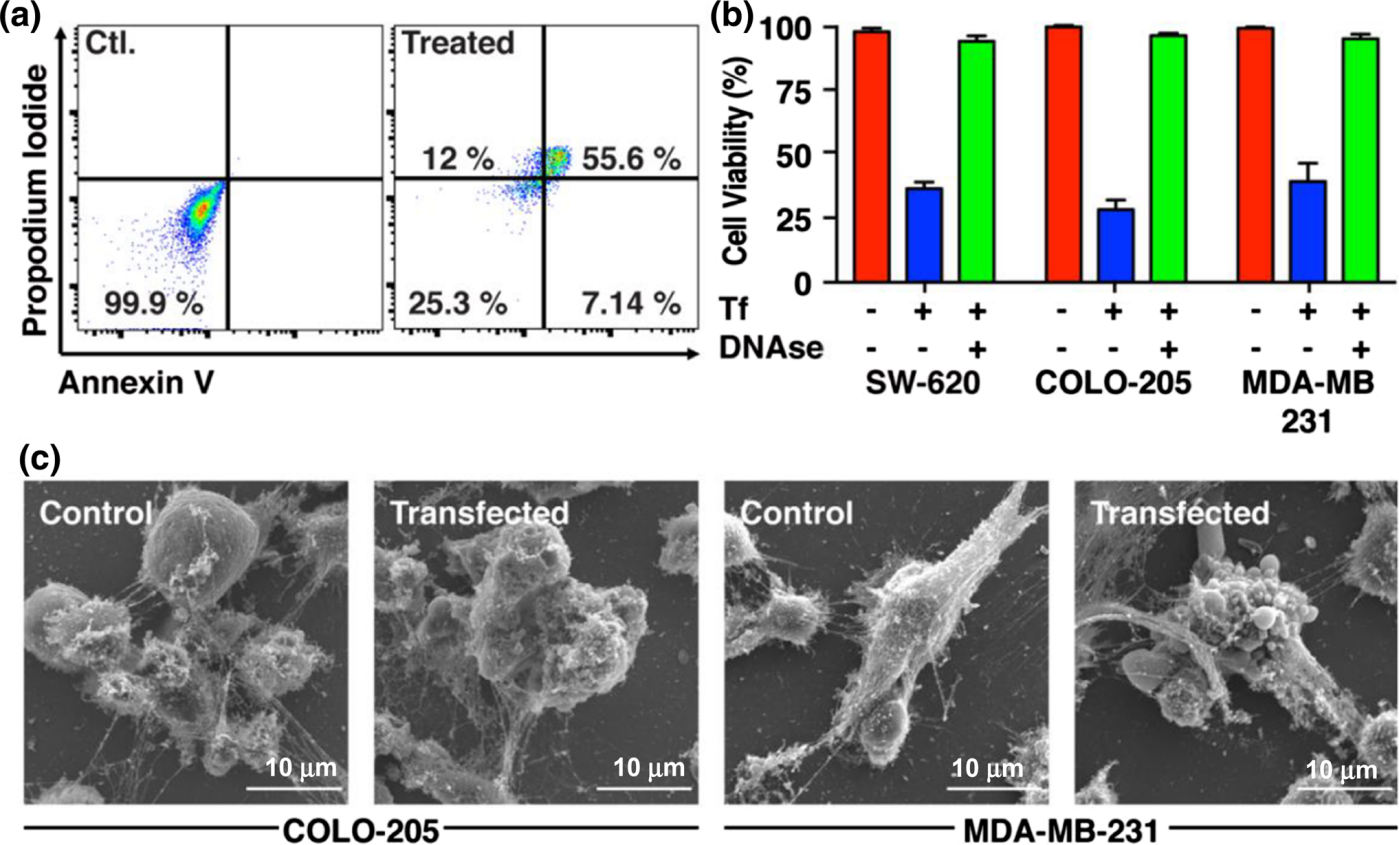
Supercharged eGFP-TRAIL expressing neutrophils trap and destroy tumor cells during NETosis. (a) Representative flow cytometry result of COLO-205 cells co-cultured with eGFP-TRAIL expressing neutrophils for 24 h and stained for apoptosis and necrosis using Annexin V and propodium iodide, respectively. (b) Cell viability quantification of indicated cancer cell lines co-cultured with wildtype neutrophils or cells positive for eGFP-TRAIL (Tf). (c) Immuno-gold SEM images of indicated cancer cells co-cultured with neutrophils.

PLB-985 derived neutrophils expressing NETs decorated with eGFP-TRAIL were co-cultured with multiple human tumor cell lines and showed significant apoptosis-inducing potential. In COLO-205, after 16 h of co-culture, 7 and 12% of the tumor cells underwent early and late apoptosis, respectively, while over 55% of the population were classified as necrotic (Fig. 7a). In the presence of eGFP-decorated NETs, we observed a > 60% kill rate of cancer cells in the three cell lines SW620, COLO 205 and MDA-MB-231 (Fig. 7b). SEM images clearly showed significant NETosis when neutrophils were co-cultured with tumor cells. However, only when NETs were decorated with eGFP-TRAIL did the tumor cells undergo apoptosis as evident by membrane blebbing (Fig. 7c).

## Conclusions

The constant presence of neutrophils near tumor cells in all stages of cancer development and in conjunction with the unique ability of neutrophils to undergo NETosis in the presence of cancer cells, make neutrophils a promising candidate for the delivery of cancer therapeutics. Here we introduced a novel form of cancer therapy by leveraging these unique aspects of neutrophils by re-engineering the cell to express a chimeric eGFP-TRAIL protein. This form of treatment exemplifies the potential of employing neutrophils as a drug delivery vehicle that, until now, has been largely unexplored.

## Methods

### Cell Culture and Differentiation

The acute myeloid leukemia PLB-985 cell line (derivative of HL-60) stably expressing Cas9 protein was a generous gift from the Collins Lab (University of California, Davis). The cells were cultured in RPMI 1640 media supplemented with 2 mM l-glutamine, 25 mM HEPES, 10% (v/v) FBS, and 2 μg/mL Blastincidin at 37 °C and 5% CO_2_. Cultured cells were regularly tested for mycoplasma using the Universal Mycoplasma Detection Kit (ATCC 30-1012K).

Neutrophil differentiation of logarithmically growing PLB-985 cells was induced by reduction of FBS to 5% and supplementation of 0.5% (vol/vol) DMF. After 3 days, an equivalent of the initial volume of differentiating medium was added and the differentiation continued until day 7.

### Plasma Cell Free DNA Isolation and Quantification

Plasma was isolated from whole blood of cancer patients and healthy donors by centrifugation, collected after informed consent. To 1 mL of plasma, 100 μL of Tris-HCl (pH 8.0) buffer containing 250 mM EDTA, 750 mM NaCl, 100 g/L of sodium dodecyl sulfate and 20 mg/mL of proteinase K was added and incubated for 2 h at 56 °C. Next, proteins were precipitated with 200 μL of 6 M NaCl solution. The cell-free DNA (cfDNA) was extracted from the supernatant using phenol-chloroform-isoamyl alcohol (25:24:1) followed by ethanol precipitation. DNA concentration was quantified using a Nano-Drop instrument.

### Vector Design

pAAVS1-eGFP-TRAIL was constructed starting with the plasmid template of pMK232 (Addgene 72834) containing the AAVS1 homology arms. The eGFP-TRAIL sequence was inserted downstream of the CMV promoter. pX330-U6-AAVS1 was constructed using the plasmid template of pX330 (Addgene 42230). The sgRNA oligonucleotides targeting the AAVS1 region were inserted into the plasmid downstream of the U6 promoter using restriction enzymes AgeI and EcoRI.

Supercharged eGFP-TRAIL DNA sequences were constructed by using site-directed mutagenesis (Agilent) PCR. Indicated amino acids of the wildtype eGFP sequence were replaced with neutral or positively charged amino acids. Protein surface charge was calculated by summing up the total charge of the eGFP sequence based on the predicted charge of the amino acids in the sequence. All plasmid constructs were verified by sequencing.

### CRISPR/CAS9 Knock-In of eGFP-TRAIL

2x10^6^ PLB-985 cells were nucleofected (AMAXA Cell Nucleofector Kit V and Amaxa Nucleofactor II, program C-023) With 20 μg of pAAVS1-eGFP-TRAIL and 20 μg of pX330-U6-AAVS1. Immediately following nucleofection, 500 μL of medium was added to the cuvette and the cells incubated at room temperature for 10 min. The cells were then cultured for 48 h. After 48 h, GFP-expressing cells were selected with culture medium supplemented with 2 μg/mL Puromycin.

### Chemical and Antibodies

TACS® Annexin V Kit (Gaithersburg, MD, USA) was used for assaying cell apoptosis. Reagents for SEM were obtained from Electron Microscopy Sciences (Hatfield, PA, USA): glutaraldehyde, osmium tetroxide and uranyl acetate. Antibodies for human TRAIL were purchased from Biolegend (San Diego, CA, USA). Primary antibodies for human TRAIL and β-actin were obtained from PeproTech (Rocky Hill, NJ, USA) and Santa Cruz Biotech (Santa Cruz, CA, USA). HRP-conjugated anti-mouse and anti-rabbit antibodies were obtained from Santa Cruz Biotech. IL-8 human Elisa Kits were purchased from ThermoFisher.
